# Heart rate variability in healthy term newborns is related to delivery mode: a prospective observational study

**DOI:** 10.1186/s12884-018-1900-4

**Published:** 2018-06-27

**Authors:** Marek Kozar, Ingrid Tonhajzerova, Michal Mestanik, Katarina Matasova, Mirko Zibolen, Andrea Calkovska, Kamil Javorka

**Affiliations:** 1 Department of Neonatology, University Hospital in Martin, Jessenius Faculty of Medicine in Martin, Comenius University in Bratislava, Martin, Kollarova 2, 03659 Martin, Slovakia; 2 Department of Physiology, Jessenius Faculty of Medicine in Martin, Comenius University in Bratislava, Mala Hora 4C, 03601 Martin, Slovakia; 3 Biomedical Center Martin, Jessenius Faculty of Medicine in Martin, Comenius University in Bratislava, Mala Hora 4C, 03601 Martin, Slovakia

**Keywords:** Heart rate variability, Full-term newborn, Delivery mode, Postnatal adaptation

## Abstract

**Background:**

Early postnatal period is characterized by dramatic adaptation changes of cardiovascular and respiratory systems in newborns. There is still insufficient data regarding maturation of autonomic regulatory mechanisms in neonates early after delivery. Aim of this study was to analyze cardiac autonomic regulation in newborns within the first few postnatal days in relation to different modes of delivery using time and spectral heart rate variability analysis.

**Methods:**

Eutrophic healthy term newborns (*n* = 46) were divided into three groups according to the delivery mode: vaginal delivery (VD group; *n* = 16), vaginal delivery with epidural analgesia (EDA group; *n* = 16), and caesarean section under general anesthesia (CS group; *n* = 14). Heart rate variability (HRV), blood pressure (BP), and blood oxygen saturation (SpO_2_) were measured within the first two hours after birth and on the third to fourth postnatal day. HRV parameters were evaluated in the time domain (RR intervals, mean square of successive differences – MSSD) and frequency domain (total spectral power – TP, absolute and relative low and high frequency powers).

**Results:**

The HRV spectral analysis showed significantly higher relative power of the high-frequency band (HF%) in the VD group compared to the CS group early after delivery (*p* = 0.002). HRV parameters and BP significantly increased on the third to fourth postnatal day in all groups (*p* < 0.05). No significant differences in basic characteristics, BP and SpO_2_ were identified between groups during both measurements.

**Conclusions:**

HRV analysis revealed higher cardiovagal modulation in spontaneously born newborns without analgesia compared to neonates born by caesarean section. It could represent a potential pathomechanism that leads to discrete abnormal neurocardiac regulation associated with higher risk for worsened postnatal adaptation of cardiovascular system in surgically delivered neonates.

## Background

The neonatal period is a vulnerable age characterized by dramatic physiological changes. The cardiovascular and respiratory systems play crucial roles in early postnatal adaptation after delivery [1]. Postnatal cardiovascular adaptation starts within the first few minutes of the onset of spontaneous breathing, but the complex process continues for several hours to days [1]. In this context, study of the cardiovascular regulatory mechanisms could provide important information about early postnatal adaptation.

The heart rate is primarily controlled through the sympathetic and parasympathetic parts of the autonomic nervous system. Newborns have high heart rate, which corresponds to cardiac-linked sympathetic predominance associated with decreased vagal activity. Thus, the development of the cardiac autonomic innervation is not complete after birth [2]. Heart rate variability (HRV) describes the oscillation in the interval between consecutive heartbeats as well as the oscillations between consecutive instantaneous heart rates [3]. In this context, HRV could provide a unique noninvasive tool to obtain information about the early regulatory mechanisms of the cardiac dynamic sympathovagal balance. This information could indicate physiological maturation and postnatal adaptation in newborns.

Recent studies revealed higher HRV associated with a tendency to increase within the first postnatal days in full-term neonates compared to preterm neonates [4]. In pathological conditions, reduced HRV is associated with hypoxic-ischemic encephalopathy [5], intraventricular hemorrhage [6], respiratory distress syndrome [7], persisting ductus arteriosus [8], infection and sepsis [9], hyperbilirubinemia requiring phototherapy treatment [10], etc. Therefore, HRV analysis has become a promising and sensitive tool in neonatal practice, which allows early detection of disease onset, even within several hours before its clinical manifestation [11].

The neonatal HRV is characterized by certain physiological mechanisms. Specifically, respiratory sinus arrhythmia (RSA) represents the most important mechanism influencing short-term HRV. Two major mechanisms are recognized in RSA generation. The first is a central mechanism characterized by the interaction between the respiratory and cardiomotoric centers, resulting in increased heart rate during inspiration (i.e., decreased vagal activity) and vice versa during expiration (i.e., increased vagal activity). The second is a peripheral mechanism involving lung inflation, which inhibit vagal activity during inspiration, as well as peripheral chemoreceptors that detect oscillations in blood oxygen, carbon dioxide, and pH [12].

With regard to HRV, the high-frequency band (HF) represents the cardiac parasympathetic modulation linked to RSA, while the low-frequency band (LF) is considered an index of baroreflex activity that is mediated by both the sympathetic and parasympathetic compartments of the autonomic nervous system [13, 14]. However, recent studies dispute that the LF is related to sympathetic activity, thus “the clinical significance of the association of cardiac sympathetic overactivity with LF power related indices is questionable” [15, 16]. Furthermore, in newborns, the higher respiratory rate (40–60 breaths per minute) should be taken into account in the modification and consequent extension of the HF range up to 1.0–2.0 Hz [2].

Moreover, HRV in newborns is influenced by other factors, such as heritability, maternal factors (e.g., maternal blood pressure (BP) and blood oxygen saturation (SpO_2_)), sleep phase, and potentially the delivery mode [17]. In surgically born neonates, recent studies observed reduced heart rate [18], HRV [19], BP [20], and SpO_2_ [21] early after delivery. Thus, we addressed the hypothesis that cardiac autonomic regulation indexed by HRV analysis could be related to delivery modes in newborns. The main objective was to study the cardiac autonomic regulatory mechanisms in healthy full-term newborns within the early postnatal period in relation to different modes of delivery using HRV analysis. Specifically, several factors were hypothesized to affect the cardiovascular regulation early after birth: the absence of physiological delivery mechanisms in babies born by caesarean section, the effect of general anesthesia / epidural analgesia and the difference in early postnatal management of newborns (absence of bonding in surgically delivered babies). Secondly, we aimed to assess the influence of delivery mode on BP and SpO_2_, which are physiological parameters that indicate postnatal adaptation. To the best of our knowledge, our study is the first one to evaluate HRV within the first few postnatal days in healthy eutrophic full-term newborns from the perspective of delivery modes.

Compared to previous brief report of the preliminary findings of this study in a recent review [22], the present paper provides a complete description of the evolution of HRV parameters and hemodynamic characteristics within the early postnatal period in relation to different modes of delivery.

## Methods

The study was performed from January 2013 to December 2015 at the Department of Neonatology in University Hospital in Martin, Slovakia - as the essential part of the first author’s PhD thesis [23].

### Study design

This prospective study included 46 healthy term newborns divided into three groups according to the mode of delivery. The *VD group* (vaginal delivery without analgesia) included 16 subjects (7 boys, 9 girls). The *EDA group* (vaginal delivery with epidural analgesia) consisted of 16 subjects (8 boys, 8 girls). A combination of bupivacaine (15 mg) and sufentanil (10 μg) was used for epidural analgesia. The *CS group* (caesarean section in general anesthesia) involved 14 subjects (7 boys, 7 girls). Intravenously administrated thiopental (500 mg) along with the inhaled anesthetic sevofluran were given to women who underwent caesarean sections. Only newborns with indications of elective caesarean sections were included (breech presentation, repeat caesarean section, primary maternal indication for caesarean section). Newborns in VD and EDA groups were delivered spontaneously and without the need for instrumental delivery (i.e., forceps and vacuum extractor).

The inclusion criteria were 37–40 weeks of gestation at birth, delivery without complications followed by physiological immediate postnatal adaptation (Apgar score ≥ 8 in 1 min and ≥ 9 in 5 min after birth) without signs of intrauterine hypoxia (umbilical arterial pH > 7.2 immediately after birth, physiological acid-base parameters), normotrophic status (birth weight between the 10th and 90th percentiles for appropriate gestational age according to Fenton growth charts), normothermia (core body temperature between 36.0 and 37.5 °C), and physiological respiratory rate indicating appropriate postnatal adaptation of the respiratory system (40–60 breaths per min). The exclusion criteria included pathological gravidity (preterm birth, preeclampsia, intrauterine growth restriction, etc.), maternal systemic disease (including gestational and other types of diabetes) and smoking during pregnancy. Subjects were also excluded in the event of congenital anomaly, perinatal infection, hypotension, hypoglycemia, or hyperbilirubinemia requiring phototherapy during the observed time.

### Measurements and data processing

Newborns underwent two measurements - the first was performed early after birth (between the first and second hour after birth) and the second measurement on the third to fourth postnatal day. The measured data (ECG signal for short-term HRV analysis, BP, and SpO_2_) were acquired in a supine position during the sleep period in neonates who were placed in an incubator (first measurement) or a neonatal cot covered with a blanket (second measurement). The second measurement was performed at least 30 min after feeding to minimize its effect on HRV.

### Heart rate variability processing

Three neonatal ECG electrodes were placed on the newborn’s chest to record the RR intervals using a telemetric system (Var Cor PF7, Neonatal, Dimea Olomouc, Czech Republic) with the sampling frequency 1000 Hz – signal sampling and digitization was done periodically with a period of 1 ms. The time interval for ECG recording was 20 min for each measurement. Recording of ECG signal was initiated after the period of approximately 30 min of heart rate stabilization. RR intervals were filtered by the elimination of artifacts caused by occasional arrhythmias or disturbances during ECG recordings using a recognition algorithm (automatic filtration method eliminating RR interval values differing by more than 20% from the preceding interval) and then controlled manually. Consecutively, the 300 RR intervals were used for the time and spectral HRV analysis. The calculation of the spectral HRV analysis was performed by using the fast Fourier transformation method with partially modified Coarse-graining Spectral Analysis (window length 256 samples). The algorithm provides optimum suppression of nonharmonic and noise components of analyzed signal.

For the time analysis, the evaluated HRV parameters were the mean duration of RR interval (ms) and mean square of successive differences (MSSD; ms). For the spectral analysis, the evaluated HRV parameters were the absolute and relative spectral power in the high-frequency band (HF: 0.15–1.5 Hz) and low-frequency band (LF: 0.04–0.15 Hz), as well as the total spectral power (TP: 0.04–1.5 Hz) [2]. The percentual expression of spectral power in each band (LF%, HF%) is related to total spectral power. Since there are no standard recommendations for HRV analysis in newborns [24], only HF frequency band was adapted (up to 1.5 Hz) reflecting high respiratory rate in newborns.

### Other physiological parameters

The physiological parameters BP and SpO_2_ were measured using a vital functions monitor (Carescape Monitor V100, GE Healthcare). The measurements were acquired immediately after the ECG recording using an oscillometric method for BP and Masimo pulse oximetry method for SpO_2_. Respiratory rate was measured visually using a stopwatch every 5 min during the ECG recording. Additionally, the status and clinical condition were evaluated based on the core body temperature measured using a standard neonatal thermometer, basic characteristic data (gestational age, birth weight, Apgar score), and selected laboratory parameters (acid-base parameters, glycaemia, serum bilirubin).

### Statistical analysis

Data were analyzed using the statistical software package Systat (Cranes Software International Ltd., USA). The normality of the distribution was tested using the Shapiro-Wilk test, and the variables with a non-normal distribution (MSSD, LF, HF, and TP) were logarithmically transformed. The equality of variance was tested using Levene’s test and all variables except for serum bilirubin showed equal variances across the evaluated samples.

One-way analysis of variance (ANOVA) was used for between-groups comparison of the parameters evaluated only early after birth (gestational age, birth weight, body temperature, respiratory rate, and acid-base parameters) or on the third to fourth postnatal day (glycaemia) (Table 1). The between-groups differences in serum bilirubin were analyzed using Kruskal-Wallis test (Table 1). HRV, BP, and SpO_2_ were tested for the effects of group and the postnatal age using a two-way repeated-measures ANOVA and post hoc Holm-Sidak multiple comparison test with alpha = 0.05 (Tables 2 and 4). The effect of group was entered as a between-subjects factor and the effect of postnatal age as a within-subjects factor. Data were additionally analysed using linear regression modelling with LF% or HF% early after birth as dependent variables and delivery mode group, gestational age, birth weight, respiratory rate and heart rate as candidate predictors. Delivery mode group was included as a categorical variable with categories “Vaginal delivery without analgesia”, “Vaginal delivery with epidural analgesia”, and “Caesarean section under general anesthesia”. Gestational age, birth weight, respiratory rate and heart rate were included as continuous variables (Table 3). The subsets of the variables which best contributed to prediction of the LF% and HF% were assessed using Mallows Cp statistics as best criterion. Presence of multicollinearity was checked using assessment of the variance inflation factor (VIF). A value of *p* < 0.05 was considered statistically significant. The presented data are expressed as the mean (x̅) ± standard deviation (SD), or median (M) with interquartile range (IQR).

**Table 1:** Characteristics of subjects

Variable			VD	EDA	CS
Sex	Boys	n (%)	7 (43.8)	8 (50)	7 (50)
	Girls		9 (56.2)	8 (50)	7 (50)
Gestational age	(weeks)	$\overline{x}$ ± SD	39.3 ± 1.2	39.3 ± 0.9	38.8 ± 1.4
Birth weight	(grams)		3507 ± 453	3402 ± 363	3535 ± 552
Acid - base	pH	$\overline{x}$ ± SD	7.32 ± 0.04	7.31 ± 0.05	7.33 ± 0.04
	BE (mmol/l)		−2.6 ± 1.3	−2.9 ± 1.6	−1.4 ± 1.9
	Lactate (mmol/l)		2.9 ± 1.1	3.2 ± 0.7	2.6 ± 1.3
Temperature	(° C)	$\overline{x}$ ± SD	36.6 ± 0.4	36.8 ± 0.4	36.6 ± 0.3
Respiratory rate	(/min)	$\overline{x}$ ± SD	47.6 ± 7.2	50.5 ± 5.3	49.8 ± 10.9
Glycaemia (II)	(mmol/l)	$\overline{x}$ ± SD	3.9 ± 0.8	3.9 ± 0.6	4.1 ± 0.7
Bilirubin (II)	(umol/l)	M (IQR)	152 (116–199)	159 (109–177)	120 (112–130)

*VD* spontaneous vaginal delivery, *EDA* spontaneous delivery with epidural analgesia, *CS* caesarean section, *BE* base excess, *II* 2nd measurement, $\overline{x}$ arithmetic mean, *SD* standard deviation, *M* median, *IQR* interquartile range

**Table 2:** Values of HRV parameters in time and frequency domains

	Parameters		Group		
			VD (*n* = 16)	EDA (*n* = 16)	CS (*n* = 14)
Time domain	RR_1_ (s)	$\overline{x}$ *±SD (SEM)*	0.501 ± 0.03 (0.008)	0.513 ± 0.05 (0.012)	0.498 ± 0.03 (0.008)
	RR_2_ (s)	$\overline{x}$ *±SD (SEM)*	0.573 ± 0.06 (0.015)	0.55 ± 0.05 (0.012)	0.564 ± 0.054 (0.014)
	log MSSD_1_ (ms)	$\overline{x}$ *±SD (SEM)*	4.14 ± 1.1 (0.28)	4.08 ± 1.5 (0.4)	3.49 ± 0.83 (0.22)
	log MSSD_2_ (ms)	$\overline{x}$ *±SD (SEM)*	6.42 ± 1.1 (0.28)	6.13 ± 0.96 (0.24)	6.21 ± 1.23 (0.33)
Frequency domain	log LF_1_ (ms^2^)	$\overline{x}$ *±SD (SEM)*	2.45 ± 1.05 (0.26)	2.86 ± 1.3 (0.32)	3.12 ± 1.02 (0.27)
	log LF_2_ (ms^2^)	$\overline{x}$ *±SD (SEM)*	4.89 ± 1.4 (0.35)	4.45 ± 1.46 (0.37)	5.37 ± 0.96 (0.26)
	log HF_1_ (ms^2^)	$\overline{x}$ *±SD (SEM)*	3.02 ± 0.92 (0.23)	3.18 ± 1.55 (0.39)	2.7 ± 1.1 (0.3)
	log HF_2_ (ms^2^)	$\overline{x}$ *±SD (SEM)*	5.38 ± 1.22 (0.31)	5.1 ± 0.93 (0.23)	5.2 ± 1.08 (0.29)
	LF_1_%	$\overline{x}$ *±SD (SEM)*	37.3 ± 14.7 (3.7) **	43.3 ± 19 (4.7)	59.2 ± 18.4 (4.9) **
	LF_2_%	$\overline{x}$ *±SD (SEM)*	40.2 ± 20.9 (5.2)	37.6 ± 21.3 (5.3)	54.1 ± 20.6 (5.5)
	HF_1_%	$\overline{x}$ *±SD (SEM)*	62.7 ± 14.7 (3.7) **	56.7 ± 19 (4.7)	40.8 ± 18.4 (4.9) **
	HF_2_%	$\overline{x}$ *±SD (SEM)*	59.8 ± 20.9 (5.2)	62.4 ± 21.3 (5.3)	45.9 ± 20.3 (5.4)
	log TP_1_ (ms^2^)	$\overline{x}$ *±SD (SEM)*	3.52 ± 0.91 (0.23)	3.81 ± 1.42 (0.36)	3.69 ± 0.98 (0.26)
	log TP_2_ (ms^2^)	$\overline{x}$ *±SD (SEM)*	5.96 ± 1.21 (0.3)	5.64 ± 0.98 (0.24)	6.09 ± 0.92 (0.25)

*VD* spontaneous vaginal delivery, *EDA* spontaneous delivery with epidural analgesia, *CS* caesarean section, *RR*_*i*_ duration of RR intervals, *log MSSD*_*i*_ common logarithm of mean square of succesive differences, *log LF*_*i*_ common logarithm of low-frequency power band, *log HF*_*i*_ common logarithm of high-frequency power band, *LF*_*i*_ *%* relative power of low-frequency band, *HF*_*i*_ *%* relative power of high-frequency band, *log TP*_*i*_ common logarithm of total spectral power, *s* second, *ms* milisecond, *ms*^*2*^ squared milisecond, $\overline{x}$ arithmetic mean, *SD* standard deviation, *SEM* standard error of the mean

**p value < 0.001. The parameters are indicated as *i* = 1 for the value at the 1st measurement and *i* = 2 for the value at the 2nd measurement

**Table 3:** Estimated effects of delivery mode and heart rate on relative spectral powers early after birth – regression analysis

Parameter	Coefficient	Std. error	Units	Standardised β	*p*-value
LF%					
Intercept	−87.496	30.614	–		0.007
EDA	7.609	5.278	–	0.189	0.157
CS	20.401	5.462	–	0.491	< 0.001
Heart rate	1.044	0.254	bpm^−1^	0.476	< 0.001
HF%					
Intercept	187.488	30.613	–		< 0.001
EDA	−7.609	5.278	–	−0.189	0.157
CS	−20.408	5.462	–	−0.491	< 0.001
Heart rate	−1.044	0.254	bpm^− 1^	−0.476	< 0.001

*EDA* vaginal delivery with epidural analgesia, *CS* caesarean section in general anaesthesia, bpm^− 1^: a change of the dependent parameter per a difference of heart rate one beat per minute. EDA and CS are dummy-coded and indicate the difference between EDA/CS mode of delivery and vaginal delivery without analgesia

## Results

Preliminary HRV data of our study were briefly described as a part of the extensive review concerning various factors and variables affecting HRV in newborns [22]. Despite the fact that prior work included all participants, this original study presents final results including values of HRV, BP and SpO_2_ together with the clinical characteristic data after consistent and appropriate statistical analysis comparing not only the between-groups differences of the variables, but also the evolution of the HRV parameters and hemodynamic characteristics within the early postnatal period in relation to different modes of delivery, that is not included in the previous study.

The detailed characteristics of the neonates are summarized in Table 1. No significant differences were found between the evaluated groups (VD, EDA, CS) in gestational age, birth weight, body temperature, respiratory rate, acid-base parameters, glycaemia, and serum bilirubin. The postnatal age of subjects was 1.44 ± 0.48 h, 1.77 ± 0.5 h, and 1.40 ± 0.4 h at the beginning of the first measurement, and 75.0 ± 3.2 h, 80.9 ± 12.5 h and 78.9 ± 8.5 h at the beginning of the second measurement in VD, EDA, and CS, respectively.

### Heart rate variability parameters

#### Between-groups comparison

The statistical analysis revealed a significant effect of the delivery mode (VD vs. EDA vs. CS) for the parameters LF% and HF% (*F*_[2]_ = 6.300, *p* = 0.004; *F*_[2]_ = 7.256, *p* = 0.002; respectively). No significant effect was found for other parameters. Post hoc tests revealed that early after birth, LF% was significantly higher and HF% was significantly lower in the CS group compared to the VD group (*p* = 0.003, *p* = 0.002, respectively). However, LF% and HF% in the EDA group were not significantly different from the CS and VD groups. No significant between-groups differences were found on the third to fourth postnatal day.

#### The effect of postnatal age

A significant effect of postnatal age (third to fourth postnatal day vs. early after birth) was found in the mean RR interval (*F*_[1]_ = 46.029, *p* < 0.001), logMSSD (*F*_[1]_ = 136.615, *p* < 0.001), logLF (*F*_[1]_ = 82.186, *p* < 0.001), logHF (*F*_[1]_ = 141.737, *p* < 0.001), and logTP (*F*_[1]_ = 142.573, *p* < 0.001). Post hoc tests revealed that the mean RR interval was significantly longer on the third to fourth postnatal day in all the evaluated groups (*p* < 0.001 for VD and CS, *p* = 0.014 for EDA group). LogMSSD, logLF, logHF, and logTP were also significantly higher on the third to fourth postnatal day in all the evaluated groups (*p* < 0.001 for all comparisons). No significant effect of interaction between the two factors (delivery mode vs. postnatal age) was found in the evaluated variables. The values of the HRV parameters are presented in Table 2.

#### Regression analysis

The linear regression models with only delivery mode group and heart rate best predicted LF% as well as HF% (R^2^ = 0.447, *p* < 0.001 for both). Early after birth, HRV parameter LF% showed a significant positive association with heart rate of 1.044 bpm^− 1^ (*p* < 0.001). When compared to VD group, LF% was significantly increased by 20.401 in newborns in CS group (*p* < 0.001). Parameter HF% was associated negatively with heart rate (− 1.044 bpm^− 1^, *p* < 0.001), and was significantly decreased by − 20.408 in CS group compared to VD group (*p* < 0.001). The results of regression analysis are presented in Table 3.

### Blood pressure and blood oxygen saturation

#### Between-groups comparison

ANOVA revealed a significant effect of the delivery mode (VD vs. EDA vs. CS) in preductal SpO_2_ (*F*_[2]_ = 3.529, *p* = 0.038). However, the post hoc test showed no significant differences between the groups within single measurements. No other significant effects of delivery mode were found.

#### The effect of postnatal age

The statistical analysis revealed a significant effect of postnatal age (third to fourth postnatal day vs. early after birth) for systolic, diastolic, and mean BP (*F*_[1]_ = 17.908, *p* < 0.001; *F*_[1]_ = 21.745, *p* < 0.001; *F*_[1]_ = 27.578, *p* < 0.001; respectively) and for preductal and postductal SpO_2_ (*F*_[1]_ = 8.191, *p* = 0.006; *F*_[1]_ = 14.585, *p* < 0.001; respectively). Post hoc tests revealed that systolic BP was significantly higher on the third to fourth postnatal day in the VD and CS groups (*p* = 0.001, *p* = 0.017, respectively), but not in the EDA group (*p* = 0.159). Diastolic and mean BPs were significantly higher on the third to fourth postnatal day in all groups (VD: *p* = 0.006, *p* = 0.001; EDA: *p* = 0.007, *p* = 0.005; CS: *p* = 0.021, *p* = 0.010; respectively). Postductal SpO_2_ was significantly higher on the third to fourth postnatal day in the VD and CS groups (*p* = 0.019, *p* = 0.026, respectively), but the EDA group showed only a tendency toward significantly higher values (*p* = 0.069). No significant pairwise differences were found in preductal blood oxygen saturation. No significant effect of interaction between the two factors (delivery mode vs. postnatal age) was found in the evaluated variables. The values of BP and SpO_2_ are presented in Table 4.

**Table 4:** Values of blood pressure and blood oxygen saturation

Parameters			Group		
			VD (*n* = 16)	EDA (*n* = 16)	CS (*n* = 14)
Blood pressure	_SYS_ BP_1_ (mmHg)	$\overline{x}$ *±SD (SEM)*	71.3 ± 7.7 (1.9)	74.4 ± 10.1 (2.5)	66.8 ± 7.4 (1.9)
	_SYS_ BP_2_ (mmHg)	$\overline{x}$ *±SD (SEM)*	80.9 ± 11.5 (2.9)	78.5 ± 9.5 (2.4)	74.3 ± 9.1 (2.4)
	_DIA_ BP_1_ (mmHg)	$\overline{x}$ *±SD (SEM)*	37.1 ± 6.3 (1.6)	38.3 ± 8.6 (2.1)	35 ± 5.1 (1.4)
	_DIA_ BP_2_ (mmHg)	$\overline{x}$ *±SD (SEM)*	44.2 ± 7.1 (1.8)	45.3 ± 9.6 (2.4)	41.3 ± 9.7 (2.6)
	_MEAN_ BP_1_ (mmHg)	$\overline{x}$ *±SD (SEM)*	48.6 ± 5.9 (1.5)	49.8 ± 7.4 (1.8)	45.6 ± 4.8 (1.3)
	_MEAN_ BP_2_ (mmHg)	$\overline{x}$ *±SD (SEM)*	55.9 ± 8.1 (2.0)	56.2 ± 8.0 (2.0)	51.8 ± 8.8 (2.4)
Blood oxygen saturation	_PRE_ SpO2_1_ (%)	$\overline{x}$ *±SD (SEM)*	96.9 ± 1.3 (0.3)	96.9 ± 1.3 (0.3)	97.4 ± 1.7 (0.5)
	_PRE_ SpO2_2_ (%)	$\overline{x}$ *±SD (SEM)*	97.7 ± 1.4 (0.3)	97.2 ± 1.5 (0.4)	98.4 ± 1.2 (0.3)
	_POST_ SpO2_1_ (%)	$\overline{x}$ *±SD (SEM)*	97.4 ± 1.4 (0.4)	97.2 ± 1.3 (0.3)	97.5 ± 1.7 (0.5)
	_POST_ SpO2_2_ (%)	$\overline{x}$ *±SD (SEM)*	98.5 ± 1.3 (0.3)	98.0 ± 1.7 (0.4)	98.6 ± 1.5 (0.4)

*VD* spontaneous vaginal delivery, *EDA* spontaneous delivery with epidural analgesia, *CS* caesarean section, _*SYS*_ *BP*_*i*_ systolic blood pressure, _*DIA*_ *BP*_*i*_ diastolic blood pressure, _*MEAN*_ *BP*_*i*_ mean blood pressure, _*PRE*_ *SpO2*_*i*_ preductal blood oxygen saturation, _*POST*_ *SpO2*_*i*_ postductal blood oxygen saturation, $\overline{x}$ arithmetic mean, *SD* standard deviation, *SEM* standard error of the mean, *mmHg* milimeter of mercury

The parameters are indicated as i = 1 for the value at the 1st measurement and i = 2 for the value at the 2nd measurement

## Discussion

This study investigated the potential effects of the delivery mode on cardiac autonomic regulation evaluated by the time and spectral HRV analysis. The main findings are the following: 1) Newborns born by vaginal delivery without analgesia (VD group) were characterized by significantly higher HF% compared to surgically delivered neonates (CS group). This indicates the highest cardiovagal regulation in spontaneously born neonates by physiological vaginal delivery without analgesia within the first postnatal day. 2) Significant increases in RR intervals and all HRV parameters were observed in all groups (VD, EDA, and CS) between the measurements. This indicates cardiac autonomic maturation within the third to fourth postnatal day in spontaneously delivered and surgically delivered neonates. 3) There were significant increases in BP and postductal SpO_2_ in all groups on the third to fourth postnatal day, which indicate appropriate postnatal cardiorespiratory adaptation.

Several mechanisms are assumed for these observations. Firstly, vaginal delivery is spontaneous natural way for postnatal adaptive processes. Specifically, mechanical forces (squeezing of the fetal body during passage through the birth canal) support the effective clearance of lung fluid from the airways and lungs, and delivery stress is accompanied by elevated stress hormones (particularly catecholamines) during physiological vaginal delivery [25, 26]. In contrast, these mechanisms are reduced in surgically delivered neonates, who thus have a higher risk for inadequate postnatal adaptation, that is associated with higher rates of respiratory morbidity [27], resulting in reduced HRV [28].

It seems that the absence of physiological delivery mechanisms could represent an important pathomechanism that leads to decreased HRV found in our groups of neonates born by caesarean section early after delivery. These findings are in accordance with another study that indicated higher LF compared to HF early after delivery, along with a rapid increase in both bands within the first hours of life [29]. With regard to the delivery mode, only one study compared the HRV between spontaneously and surgically born newborns, showing decreased HRV in neonates delivered by caesarean section within three days postpartum [19]. Despite the fact that these results are similar to our findings during the first measurement (significantly lower HF% in the CS group vs. the VD group), we did not identify these differences during the second measurement (on the third to fourth postnatal days). This discrepancy could be explained by unequal inclusion criteria for the CS group [19], which included neonates born by urgent caesarean section due to pathological intrauterine conditions for HRV. Nevertheless, it seems that HRV parameters could indicate subtle differences in cardiac chronotropic regulation according to the delivery mode.

Moreover, the anesthesia represents another potential factor in the HRV of surgically delivered newborns. In particular, the thiopental used in our study has an inhibitory effect on both the sympathetic and parasympathetic nervous systems [2]. Thus, we suggest a potential effect of thiopental on HRV in surgically delivered newborns in our study. In contrast, the epidural analgesia applied prior to spontaneous delivery (EDA group) did not significantly affect HRV during the first measurement, indicating a short-lasting EDA duration without significant influence on the HRV, as reported previously [30].

Consideration is warranted for the different management of newborns early after birth (skin-to-skin contact observed in spontaneously born neonates in both the VD and EDA groups). In our study, spontaneously born healthy newborns were laid on the chest of the mother immediately after birth to bond for 20 min. Higher cardiovagal regulation can result from appropriate stimulation of the receptors, including skin, olfactory and visual stimuli, during the close emotional attachment that forms between the newborn and mother [31, 32]. Thus, it seems that our findings of higher HF% in spontaneously born newborns might reflect neurophysiological mechanisms included in “skin-to-skin” attachment, which was absent in the CS group.

The lowest values of HF power observed early after delivery in the CS group could be important for future clinical observations. In particular, vagal abnormalities early after birth are associated with higher risk of neurodevelopmental disorders, such as autism [33]. Therefore, it is questionable whether the discrete HRV abnormalities in different delivery modes in this study could represent a potential mechanism leading to altered neurocardiac integrity that could result in diseases later in life, or whether they reflect a pathological sign of disorders or complications. However, this question is hypothetical and longitudinal studies are required.

In regard to early developmental changes, our results revealed significant increases in HRV in all three groups on the third to fourth postnatal day. Several studies demonstrated higher LF associated with a dominant HF increase within few days after delivery [4, 34]. We suggest that acceleration in the HRV indices within the first days after delivery could reflect the maturation of cardiovascular regulatory mechanisms, the influence of respiration leading to the amplification of respiratory sinus arrhythmia and the gradual withdrawal of postpartal stress [17].

Among other physiological parameters, our results revealed the lowest values of BP in the CS group compared to spontaneously born newborns (VD, EDA) early after birth, but the differences were not significant. Several studies demonstrated similar results of higher BP in newborns after spontaneous delivery [20, 35] as a result of longer and more intensive effects of stress hormones [36]. Studies related to the impact of delivery mode on BP development later after birth are rare. Previous works reported a persisting significant difference in BP between spontaneously and surgically delivered newborns (lower BP in the CS group) from the first few days [37] until three weeks postpartum [38]. However, a recent study did not confirm these findings [39]. Developmentally, there was a significant increase in systemic BP in all subjects on the third to fourth postnatal day, regardless of the delivery mode. This finding could potentially be the consequence of gradual stabilization of the hemodynamics and maturation of the cardiovascular regulation mechanisms. Further research with continuous recording of blood pressure is needed.

Our study did not reveal a significant difference in preductal versus postductal SpO_2_ in all groups early after birth, which contrasts with recent studies reporting higher preductal SpO_2_ [21, 40]. The higher preductal versus postductal SpO_2_ immediately after delivery could be attributed to transient pulmonary hypertension resulting in right-to-left blood shunting via the ductus arteriosus. Since SpO_2_ was measured at approximately 3 h after birth in our study, closure of the ductus arteriosus is expected in the majority of our subjects, which would result in the disappearance of the difference in pre- and postductal SpO_2_ values.

Recent modern management of deliveries should guarantee equivalent postnatal adaptation of cardiovascular and respiratory systems. However, our results are the first to reveal that the parasympathetic component of cardiac autonomic regulation is the lowest in the CS group compared to spontaneously born neonates (VD, EDA) early after birth. This finding could indicate an association between caesarean section and a higher risk of worsened postnatal cardiovascular adaptation compared to spontaneously born babies.

### Limitations of the study

The number of newborns in each group was relatively small, so further research on large groups is needed to confirm our results. Moreover, the sleep stage (active or quiet sleep) in newborns was not specified. From this perspective, research using polysomnographic recording could provide additional important information to elucidate this issue.

Regarding the HRV analysis, the physiological interpretation of the LF band is extensively discussed. Despite the fact that the previous studies indicated that LF could be used as a possible marker of cardiac sympathetic regulation [41, 42], recent evidence showed different results: 1) LF is substantially blocked by vagal, but not sympathetic blockade, 2) physiological and psychological manipulations increasing sympathetic outflow often do not raise the LF, but reduce it, 3) pharmacological interventions, e.g. infusion of isoprenaline, inducing sympathetic activation do not enhance LF, 4) there is a lack of associations between LF and valid indicators of sympathetic cardiac regulation - pre-ejection period or cardiac norepinephrine spillover [43]. This question remains controversial in the research [16], therefore, interpretation of changes in LF, as well as LF% values observed in our study is not straightforward, should be considered only with caution and the main result of this study is the finding of differences in cardiovagal regulation.

## Conclusions

This study evaluated cardiac-linked autonomic modulation by the time and spectral HRV analysis. The results revealed that the modulation was higher in newborns spontaneously born without analgesia compared to neonates born by caesarean section. We suggest that low cardiac vagal regulation in babies after surgical delivery could represent a risk factor for later postnatal adaptation.

## Abbreviations

ANOVA: Analysis of variance
BP: Blood pressure
CS: Caesarean section
EDA: Epidural analgesia
HF: High-fequency spectral power
HF%: Relative high-fequency spectral power
HRV: Heart rate variability
LF: Low-frequency spectral power
LF%: Relative low-frequency spectral power
MSSD: Mean square of successive differences
RSA: Respiratory sinus arrhythmia
SpO_2_: Blood oxygen saturation
TP: Total spectral power
VD: Vaginal delivery
VIF: Variance inflation factor
